# Exendin-4 Protected against Cognitive Dysfunction in Hyperglycemic Mice Receiving an Intrahippocampal Lipopolysaccharide Injection

**DOI:** 10.1371/journal.pone.0039656

**Published:** 2012-07-23

**Authors:** Hei-Jen Huang, Yen-Hsu Chen, Keng-Chen Liang, Yu-Syuan Jheng, Jhih-Jhen Jhao, Ming-Tsan Su, Guey-Jen Lee-Chen, Hsiu Mei Hsieh-Li

**Affiliations:** 1 Department of Nursing, Mackay Medicine, Nursing and Management College, Taipei, Taiwan; 2 Department of Life Science, National Taiwan Normal University, Taipei, Taiwan; 3 Department of Psychology, National Taiwan University, Taipei, Taiwan; Oregon Health & Science University, United States of America

## Abstract

**Background:**

Chronic hyperglycemia-associated inflammation plays critical roles in disease initiation and the progression of diabetic complications, including Alzheimer’s disease (AD). However, the association of chronic hyperglycemia with acute inflammation of the central nervous system in the progression of AD still needs to be elucidated. In addition, recent evidence suggests that Glucagon-like peptide-1 receptor (GLP-1R) protects against neuronal damage in the brain. Therefore, the neuroprotective effects of the GLP-1R agonist exendin-4 (EX-4) against hyperglycemia/lipopolysaccharides (LPS) damage were also evaluated in this study.

**Methodology/Principal Findings:**

Ten days after streptozotocin (STZ) or vehicle (sodium citrate) treatment in mice, EX-4 treatment (10 µg/kg/day) was applied to the mice before intrahippocampal CA1 injection of LPS or vehicle (saline) and continued for 28 days. This study examined the molecular alterations in these mice after LPS and EX4 application, respectively. The mouse cognitive function was evaluated during the last 6 days of EX-4 treatment. The results showed that the activation of NF-κB-related inflammatory responses induced cognitive dysfunction in both the hyperglycemic mice and the mice that received acute intrahippocampal LPS injection. Furthermore, acute intrahippocampal LPS injection exacerbated the impairment of spatial learning and memory through a strong decrease in monoaminergic neurons and increases in astrocytes activation and apoptosis in the hyperglycemic mice. However, EX-4 treatment protected against the cognitive dysfunction resulting from hyperglycemia or/and intrahippocampal LPS injection.

**Conclusions/Significance:**

These findings reveal that both hyperglycemia and intrahippocampal LPS injection induced cognitive dysfunction via activation of NF-κB-related inflammatory responses. However, acute intrahippocampal LPS injection exacerbated the progression of cognitive dysfunction in the hyperglycemic mice via a large increase in astrocytes activation-related responses. Furthermore, EX-4 might be considered as a potential adjuvant entity to protect against neurodegenerative diseases.

## Introduction

Chronic hyperglycemia-associated inflammation is considered to play critical roles in disease initiation and the progression of diabetic complications including Alzheimer’s disease (AD). Evidence has shown that diabetes mellitus (DM) increases the risk of dementia by 50–100% for sporadic Alzheimer's disease (SAD) and 100–150% for vascular dementia [1]. *In vitro* evidence has also demonstrated that chronic hyperglycemia enhances lipopolysaccharide (LPS)-elicited gene expression involved in inflammation and tissue destruction [2]. Evidence has further suggested that metabolic syndrome associated with elevated serum levels of inflammation accelerates cognitive aging [3]. However, controversial results regarding the effect of anti-inflammatory therapy on dementia were obtained in epidemiological studies and clinical trials [4], [5]. Therefore, the relationship between hyperglycemia and inflammatory response still needs to be clarified in the central nervous system.

LPS, also known as a bacterial endotoxin, is gram-negative bacterial cell surface proteoglycan and is traditionally used to stimulate an immune response. Microglia stimulated by LPS have been reported to increase many inflammation-related responses *in vitro*, including upregulation of NF-κB, IL-1β, iNOS, TLR4, COX1, and COX2 [6], [7], [8], [9], [10]. LPS injection also induces microglia and astrocyte activation [11]. Previous studies have shown that brains from AD patients were characterized by neuroinflammation, including marked astrocytosis, elevated levels of proinflammatory cytokines, and microglia activation [12], [13]. In addition, recent evidence further suggests that activation of the immune system and oxidative stress are important for the progression of AD and DM [14]. Furthermore, inflammation also induces deficits in neurotransmitter-releasing neurons such as cholinergic, noradrenergic, and serotonergic neurons [15], [16], [17], [18]. Therefore, analysis of the multivariate response to LPS will lead to a better understanding of neuroinflammatory diseases.

AD is a multifactor disorder; therefore, the development of effective interventions for patients with AD has been challenging. Recent study has suggested that EX-4 administered peripherally not only improves metabolic parameters but also induces beneficial effects on cognitive function [19]. EX-4 is a long-acting analogue of glucagon-like peptide-1 (GLP-1), which is one of the incretin hormones secreted from intestinal L cells [20]. In addition, evidence shows that EX-4 is able to cross the blood–brain barrier easily [21], and GLP-1 receptors are also highly expressed on neurons [22], [23]. Therefore, the effects of EX-4 treatment on cognitive function related to metabolism dysfunction and inflammation were further evaluated in this study.

## Results

### Hyperglycemia was Induced by STZ, but EX-4 Reduced Blood Glucose

**Figure 1:**
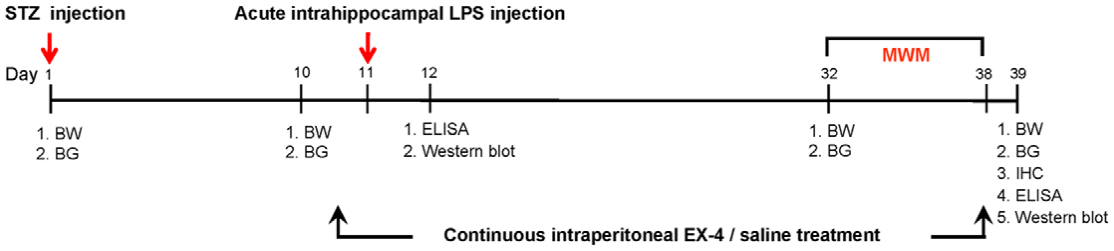
The experimental timeline of this study. Body weight (BW) and blood glucose (BG) were measured on days 1, 10, 32, and 39. ELISA, western blot, and immunohistochemistry (IHC) were performed for protein analysis. Spatial learning and memory was evaluated by the Morris water maze (MWM).

**Figure 2:**
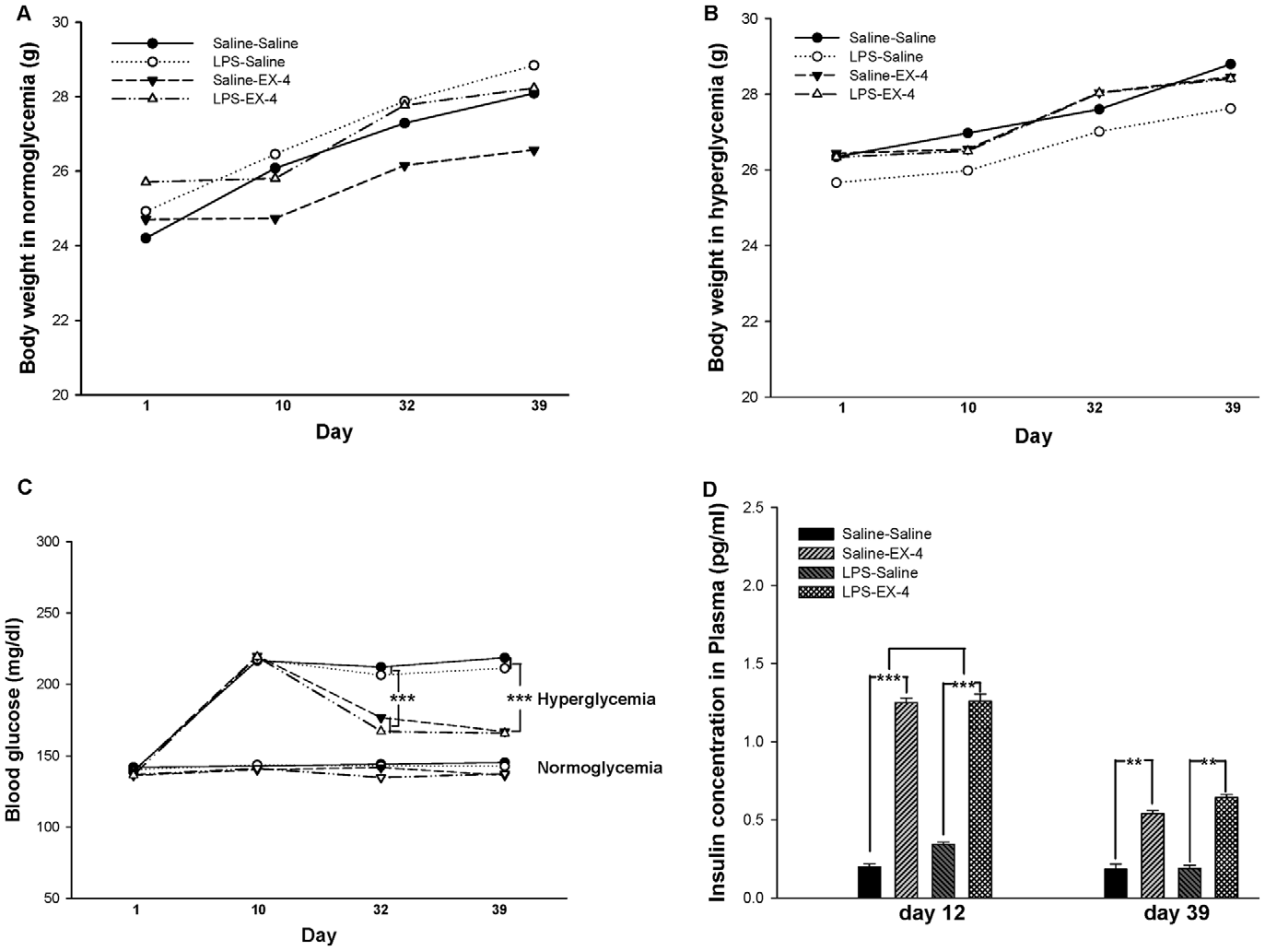
The body weight, blood glucose, and insulin level of the mice. Gain in body weight was not affected by LPS or EX-4 treatment in the normoglycemic (A) and hyperglycemic (B) mice. EX-4 treatment continuously decreased blood glucose (C) and increased insulin (D) in the hyperglycemic mice. Data are expressed as means ± S.E.M. (*n = *15–20 per group for body weight and blood glucose, *n = *6 per group for insulin). * *p*<0.05; ** *p*<0.01; *** *p*<0.001.

In order to assess the effects of EX-4 in normoglycemic and hyperglycemic mice treated with an intrahippocampal LPS/saline injection, we set up a mouse treatment timeline, as shown in Fig. 1. First, mouse body weight was measured at different time points during the experimental procedure. A gain of body weight was observed in the normoglycemic mice (F(4,64) = 13.656, *p*<0.001; Fig. 2A), whether intrahippocampal LPS injection (F(4,64) = 12.808, *p*<0.001; Fig. 2A) was associated with EX-4 treatment (F(4,94) = 5.987, *p*<0.001; Fig. 2A) or saline treatment (F(4,94) = 3.713, *p*<0.01; Fig. 2A). The results regarding body weight in the hyperglycemic mice were similar to those in the normoglycemic mice (Fig. 2B). Furthermore, blood samples collected from the mouse tail at different time points showed that blood glucose was significantly increased after acute high-dose STZ treatment, (Fig. 2C), whether with intrahippocampal LPS (F(1,140) = 296.8110, *p*<0.001) or saline injection (F(1,140) = 236.3679, *p*<0.001), at different time points (*p*<0.001), and an STZ × different time points interaction was observed (*p*<0.001). In addition, intrahippocampal LPS injection did not change the blood glucose level of mice with normoglycemia or hyperglycemia (*p*>0.05). However, EX-4 treatment decreased the blood glucose level at day 32 (F(1,55) = 51.4120, *p*<0.001) and day 39 (F(1,55) = 101.9126, *p*<0.001) only in the hyperglycemic mice. There was no interaction of LPS × EX-4 in the normoglycemic or hyperglycemic mice (*p*>0.05). Therefore, these results show that an acute high dose of STZ induced a chronic hyperglycemic condition, while EX-4 treatment ameliorated hyperglycemia and had no effect on normoglycemia.

### EX-4 Treatment Increased the Levels of the Insulin in the Plasma of Hyperglycemic Mice

We further examined the levels of insulin in the plasma of the mice (Fig. 2D). EX-4 treatment significantly increased the level of insulin in the hyperglycemic mice on day 12 (F(1,11) = 14.664, *p*<0.001) and day 39 (F(1,11) = 6.5411, *p*<0.01). In addition, there was no effect of EX-4 on the insulin level of the normoglycemic mice (data not shown). Therefore, these results suggested that EX-4 treatment increased the level of insulin in the hyperglycemic mice.

### Intrahippocampal LPS Injection Exacerbated the Impairment of Spatial Learning and Memory caused by Hyperglycemia, but EX-4 Treatment Protected against the Impairment

**Figure 3:**
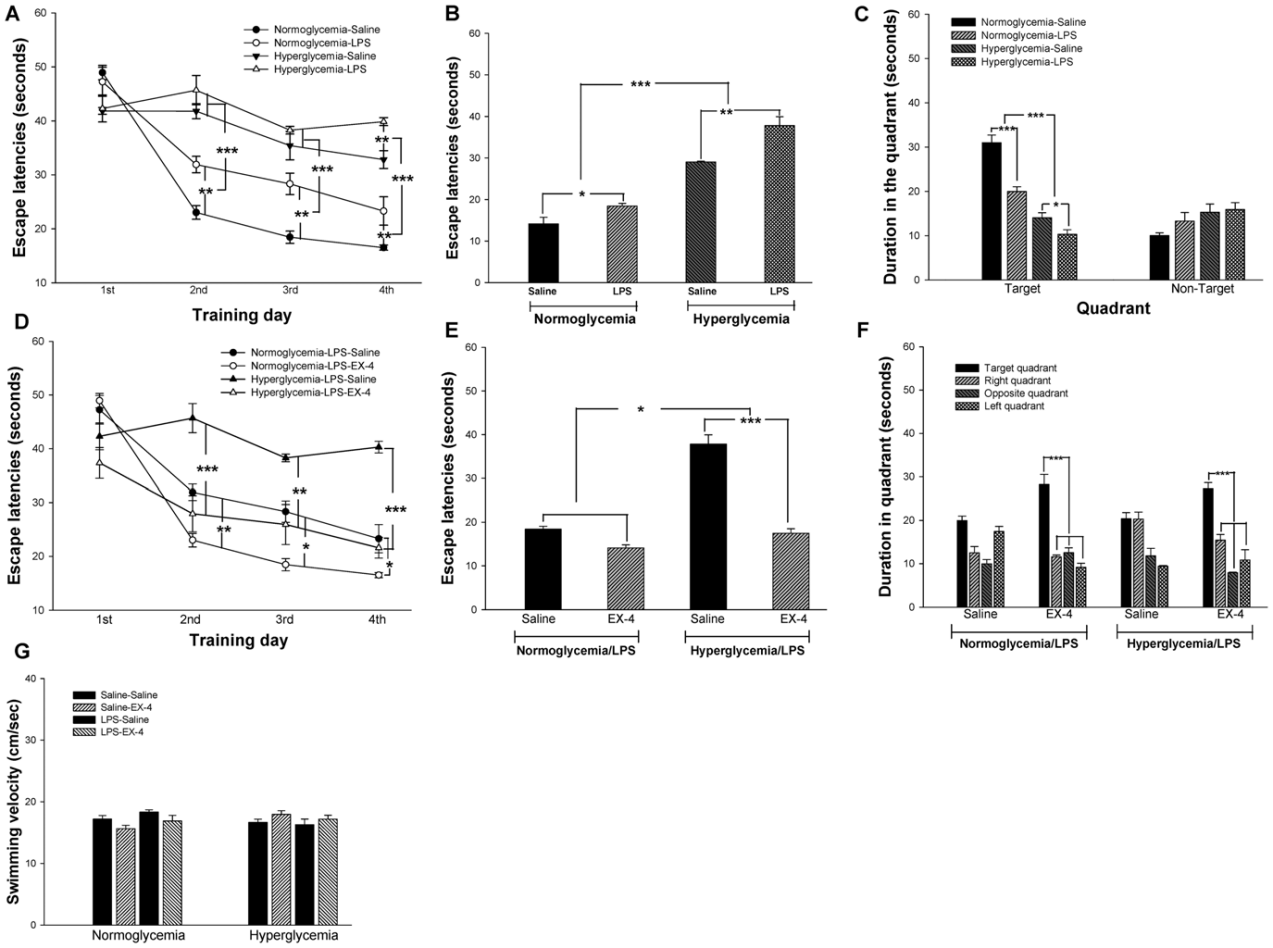
Effects of hyperglycemia, LPS and EX-4 on spatial reference learning and memory. The mouse learning curve is shown in (A). Intrahippocampal LPS injection exacerbated the impairment of spatial learning acquisition (B) and spatial memory retrieval (C) in the hyperglycemic mice. In addition, EX-4 treatment protected against the impairment of spatial learning acquisition (D & E) and spatial memory retrieval (F) following intrahippocampal LPS injection in the normoglycemic or hyperglycemic mice. These results were independent of the swimming velocity (G). Data are expressed as means ± S.E.M. (*n = *15–20 per group). * *p*<0.05; ** *p*<0.01; *** *p*<0.001.

On day 32, spatial reference learning and memory were evaluated by the Morris water maze (MWM). First, the effect of hyperglycemia and intrahippocampal LPS injection on spatial reference learning and memory was evaluated. During the training period, the latency to reach the platform in the 4 trials of each training day was averaged and assessed as the spatial learning ability (Fig. 3A). Our results demonstrated that the hyperglycemic mice spent more time searching for the platform on training day 2 (F(1,15) = 79.8681, *p*<0.001), day 3 (F(1,15) = 57.2786, *p*<0.001) and day 4 (F(1,15) = 102.0365, *p*<0.001) as compared with the normoglycemic mice (Fig. 3A). In addition, intrahippocampal LPS injection also significantly increased the escape latency as compared with intrahippocampal saline injection on training day 2 (F(1,15) = 12.3008, *p*<0.01; Fig. 3A), day 3 (F(1,15) = 12.9729, *p*<0.01; Fig. 3A) and day 4 (F(1,15) = 18.2048, *p*<0.01; Fig. 3A). No hyperglycemia × LPS interaction was identified on any training days (*p*>0.05). However, *post-hoc* multiple analysis revealed that the hyperglycemic mice that received an intrahippocampal LPS injection spent significantly more time searching for the platform than those that received saline treatment on training day 4 (*p*<0.01; Fig. 3A). From the above results, we found that a deficit in spatial learning ability was present in the mice with hyperglycemia and those that underwent intrahippocampal LPS injection. In addition, intrahippocampal LPS injection enhanced the deficit in the spatial learning ability of the hyperglycemic mice.

During the test period, the time spent searching for the platform was assessed as the acquisition of spatial learning. The escape latencies of the hyperglycemic mice were significantly increased as compared with the normoglycemic mice (F(1,11) = 153.7506, *p*<0.001; Fig. 3B). In addition, the escape latencies of the mice that received an intrahippocampal LPS injection were significantly increased as compared with the mice that received a saline injection (F(1,11) = 22.2512, *p*<0.01; Fig. 3B). There was no hyperglycemia × LPS interaction during the testing period (*p*>0.05). *Post-hoc* analyses further showed that intrahippocampal LPS injection significantly increased the escape latency in the hyperglycemic mice (*p*<0.01; Fig. 3B).

During the probe trial, the time spent in the target quadrant was assessed as the retrieval of spatial memory. The time spent in the target quadrant was reduced in the mice that received an intrahippocampal LPS injection (F(1,100) = 22.9610, *p*<0.001; Fig. 3C) and in those with hyperglycemia (F(1,100) = 84.7731, *p*<0.001; Fig. 3C). An interaction between LPS injection and hyperglycemia was observed (F(1,100) = 22.9610, *p*<0.001; Fig. 3C). Therefore, the above results suggest that intrahippocampal LPS injection exacerbated the impairment of spatial learning and memory in the hyperglycemic mice.

The effect of EX-4 treatment on these mice was evaluated at the same time, and the results showed that EX-4 reduced the time searching for the platform in the hyperglycemic mice that received an intrahippocampal LPS injection on training day 2 (*p*<0.001; Fig. 3D), day 3 (*p*<0.01; Fig. 3D), and day 4 (*p*<0.001; Fig. 3D). EX-4 treatment also decreased the platform searching time for the normoglycemic mice that received an intrahippocampal LPS injection on training day 2 (*p*<0.01), day 3 (*p*<0.05) and day 4 (*p*<0.05) (Fig. 3D). During the test period, the hyperglycemic mice that received an intrahippocampal LPS injection spent more time searching for the platform as compared with the normoglycemic mice that received an intrahippocampal LPS injection (F(1,11) = 9.9542, *p*<0.05; Fig. 3E). Furthermore, EX-4 treatment rescued the impairment of acquisition of spatial reference learning in the hyperglycemic mice that received an intrahippocampal LPS injection (F(1,11) = 47.3665, *p*<0.001; Fig. 3E). There was no interaction between hyperglycemia, intrahippocampal LPS injection and EX-4 in the acquisition of spatial reference learning (*p*>0.05). During the probe trial, EX-4 treatment increased the retrieval of spatial memory in the LPS-injected mice with normoglycemia (F(3,17) = 39.216, *p*<0.001; Fig. 3F) and hyperglycemia (F(3,17) = 31.328, *p*<0.001; Fig. 3F).

Furthermore, there was no significant difference in the swimming velocity between these groups of mice (*p*>0.05; Fig. 3G). Therefore, these findings suggest that acute intrahippocampal LPS injection exacerbated the impairment of spatial reference learning and memory in the hyperglycemic mice, and EX-4 treatment protected against the impairment.

### EX-4 Treatment Inhibited the Upregulation of IL-1β in the Hyperglycemic Mice

The levels of IL-1β in the mouse peripheral and central systems were measured 12 hr after intrahippocampal LPS or saline injection (day 12) and after the MWM (day 39) (Table 1). On day 12, the levels of IL-1β in the hippocampus were significantly different between the hyperglycemia (F(1,34) = 24.7594, *p*<0.001), intrahippocampal LPS injection (F(1,34) = 46.3172, *p*<0.001), EX-4 treatment (F(1,34) = 8.7771, *p*<0.01), hyperglycemia × intrahippocampal LPS injection (F(1,34) = 5.1498, *p*<0.05), and hyperglycemia × intrahippocampal LPS injection × EX-4 treatment groups (F(1,34) = 4.3447, *p*<0.05). In addition, intrahippocampal LPS injection also significantly increased the level of IL-1β in the plasma (F(1,50) = 6.0624, *p*<0.05). However, EX-4 treatment transiently increased the level of IL-1β in the hippocampus of the normoglycemic mice and decreased the level in the hyperglycemic mice (*p*<0.05). On day 39, IL-1β was not detected in the hippocampus. In addition, IL-1β was not detected in the plasma after EX-4 treatment. These results show that the IL-1β level was transiently increased in the hippocampus of the hyperglycemic mice. In addition, intrahippocampal LPS injection transiently increased the IL-1β levels in the plasma and hippocampus of the mice. Furthermore, intrahippocampal LPS injection enhanced the level of IL-1β in the hippocampus of the hyperglycemic mice. However, EX-4 treatment immediately inhibited the upregulation of IL-1β in the hyperglycemic mice and transiently increased the IL-1β level in the normoglycemic mice that received either an intrahippocampal LPS or a saline injection.

**Table 1:** The levels of IL-1β in the plasma and hippocampus of each group at different time points.

	Day 12		Day 39	
	Plasma(pg/ml)	Hippocampus(pg/µg)	Plasma(pg/ml)	Hippocampus(pg/µg)
N/S/S	33.44±2.01	0.90±0.05	6.57±0.54	ND
N/S/E	28.44±2.59	38.09±6.23^c^ *	ND	ND
N/L/S	32.69±3.99	30.97±5.35^b^ ***	9.73±1.63	ND
N/L/E	46.99±2.74^b*^	194.12±2.58^b^ *** ^,^ c *	ND	ND
H/S/S	29.80±4.94	113.76±4.58^a^ ***	5.02±0.25	ND
H/S/E	37.55±6.24	103.02±7.19^a^ *** ^,^ c **	ND	ND
H/L/S	50.98±5.51^b*^	341.30±7.25^a^ *** ^,^ b ***	13.91±0.65	ND
H/L/E	38.91±2.16	297.29±7.89^a^ *** ^,^ b *** ^,^ c **	ND	ND

Each value represents the mean ± SEM (n = 5–6 for each group).

a Normoglycemia compared with hyperglycemia.

b Saline compared with LPS.

c Saline compared with EX-4.

ND indicates not detected.

*p*<0.05;

** *p*<0.01;

*** *p*<0.001.

### EX-4 Treatment Increased the MnSOD Level and Reduced the Levels of COX1, COX2, CD45, and NF-κB

**Figure 4:**
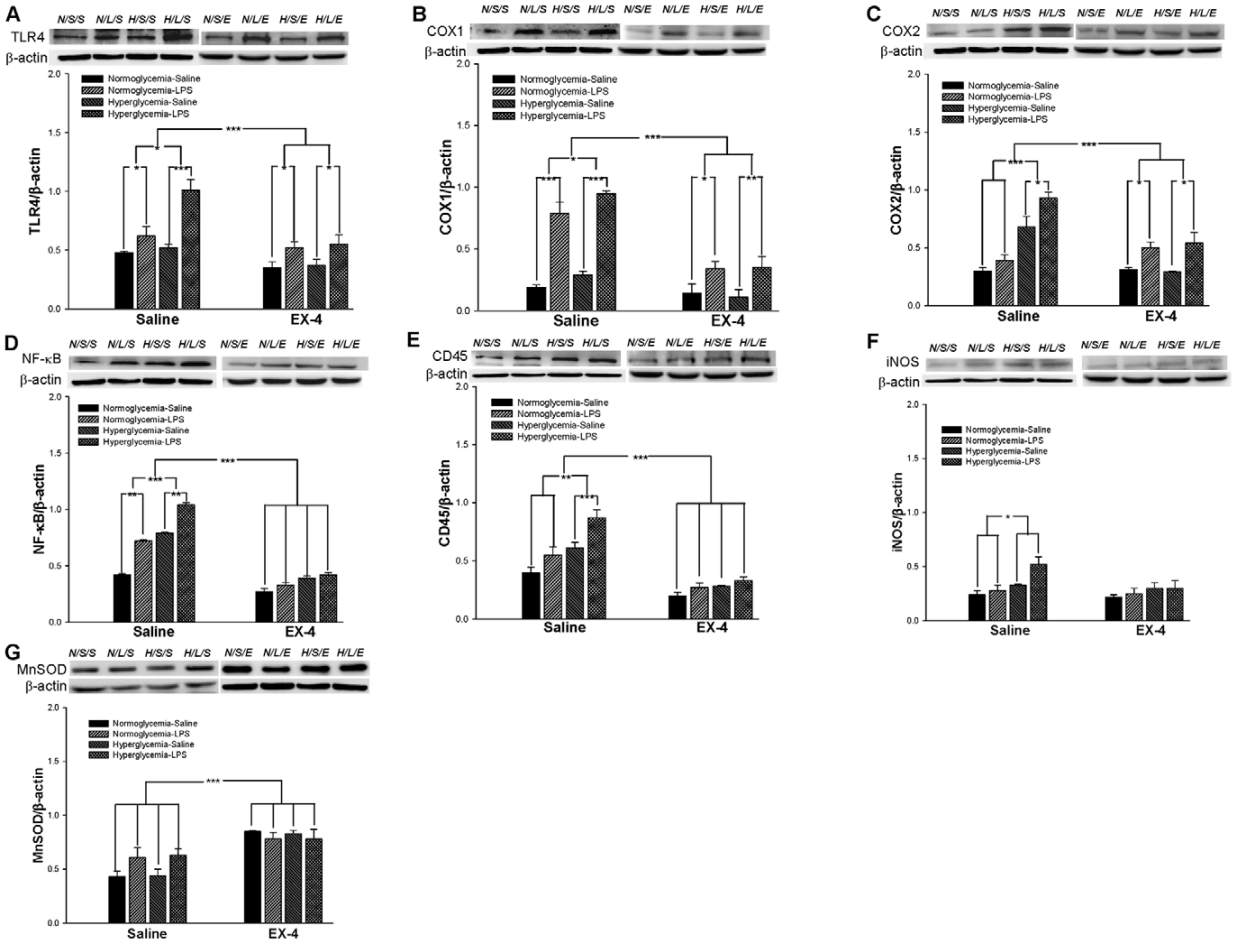
The levels of inflammation and oxidative stress in the mouse hippocampus on day 12. The expression levels of TLR4 (A), COX1 (B), COX2 (C), NF-κB (D), CD45 (E), iNOS (F), and MnSOD (G) in the mouse hippocampus were measured by western blot analyses. Data are normalized to β-actin and expressed as the mean ± S.E.M. (*n = *3–5 per group). * *p*<0.05; ** *p*<0.01; *** *p*<0.001.

**Figure 5:**
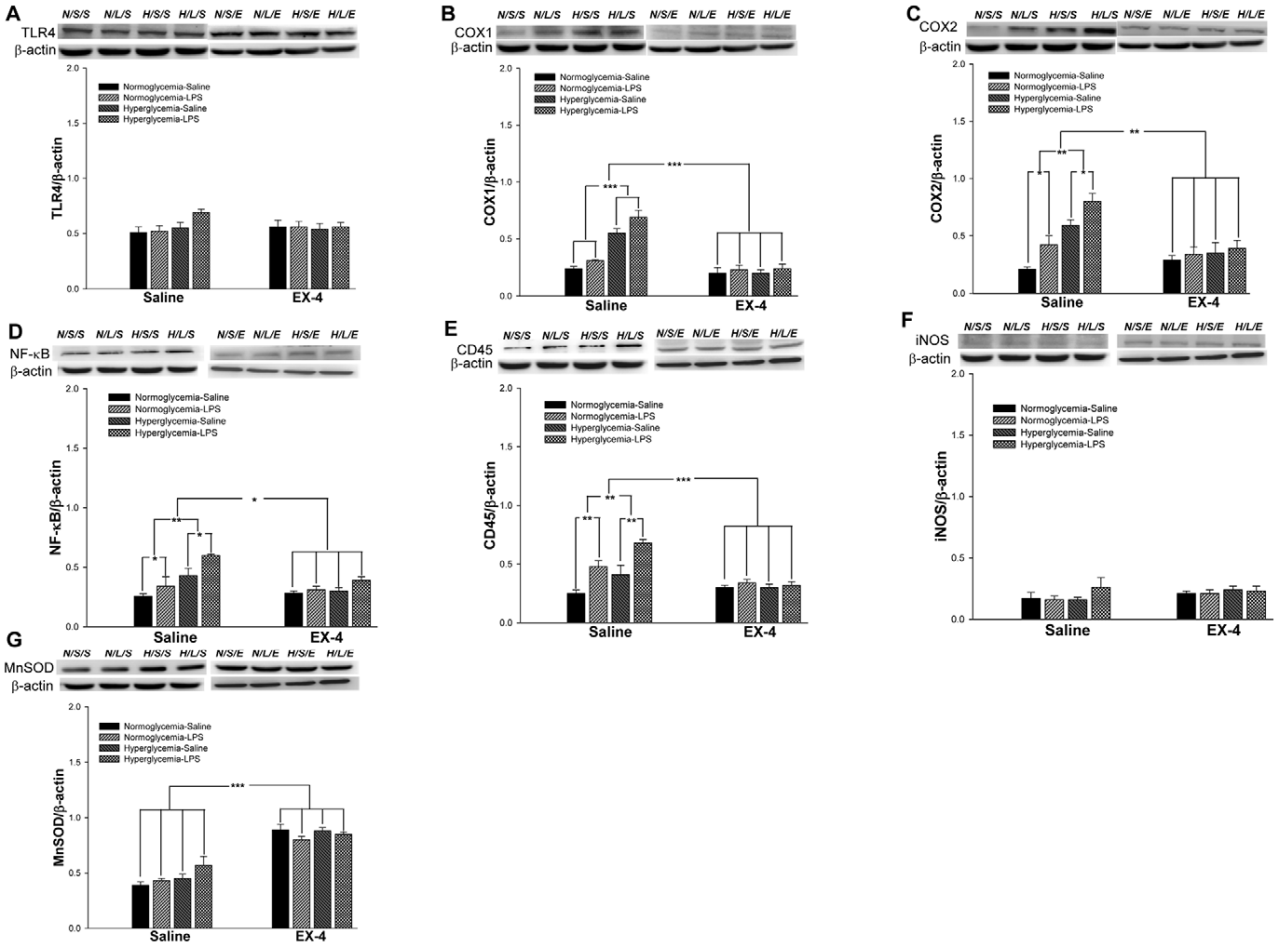
The levels of inflammation and oxidative stress in the mouse hippocampus on day 39. The expression levels of TLR4 (A), COX1 (B), COX2 (C), NF-κB (D), CD45 (E), iNOS (F), and MnSOD (G) in the mouse hippocampus were measured by western blot analyses. Data are normalized to β-actin and expressed as the mean ± S.E.M. (*n = *3–5 per group). * *p*<0.05; ** *p*<0.01; *** *p*<0.001.

Molecules related to inflammation and oxidative stress were examined at days 12 (Fig. 4) and 39 (Fig. 5), respectively, to understand the progression of pathogenic characterization under hyperglycemic and LPS conditions. The quantitative results are summarized in Table 2.

**Table 2:** The responses of inflammatory-related proteins in the hippocampus of mice receiving different treatments at different time points.

	Day 12			Day 39		
	Hyperglycemia	LPS	EX-4	Hyperglycemia	LPS	EX-4
TLR4	*a*↑	*b*↑↑↑	*c*↓↓↓			
COX1	*a*↑	*b*↑↑↑	*c*↓↓↓, *e*↓ ↓↓	*a*↑↑↑		*c*↓↓↓, d↓ ↓↓
COX2	*a*↑↑↑	*b*↑↑	*c*↓↓↓, d↓ ↓↓	*a*↑↑	*b*↑	*c*↓↓, d↓ ↓
NF-κB	*a*↑↑↑	*b*↑↑	*c*↓↓↓, d↓ ↓, *e*↓	*a*↑↑	*b*↑	*c*↓, d↓
CD45	*a*↑↑↑	*b*↑↑	*c*↓↓↓, d↓	*a*↑↑	*b*↑↑	*c*↓↓↓, d↓ ↓, *e*↓↓↓
iNOS	*a*↑					
MnSOD			*c*↑↑↑			*c*↑↑↑

a Hyperglycemia compared with normoglycemia.

b LPS compared with saline.

c EX-4 compared with saline.

d hyperglycemia × EX-4.

e LPS × EX-4.

↑Increased (*p*<0.05); ↑↑ increased (*p*<0.01); ↑↑↑ increased (*p*<0.001).

↓Decreased (*p*<0.05); ↓↓ decreased (*p*<0.01); ↓↓↓ decreased (*p*<0.001).

The results of western blot analysis from day 12 indicated that LPS injection increased the expression levels of TLR4 (F(1,23) = 27.5134, *p*<0.001; Fig. 4A ), COX1 (F(1,23) = 86.1217, *p*<0.001; Fig. 4B), COX2 (F(1,23) = 23.3820, *p*<0.001; Fig. 4C), NF-κB (F(1,23) = 13.1911, *p*<0.01; Fig. 4D), and CD45 (F(1,23) = 12.2750, *p*<0.01; Fig. 4E). In addition, the levels of TLR4 (F(1,23) = 6.5066, *p*<0.05; Fig. 4A), COX1 (F(1,23) = 1.5703, *p*<0.05; Fig. 4B), COX2 (F(1,23) = 33.3854, *p*<0.001; Fig. 4C), NF-κB (F(1,23) = 23.8678, *p*<0.001; Fig. 4D), CD45 (F(1,23) = 20.11113, *p*<0.001; Fig. 4E), and iNOS (F(1,23) = 6.8425, *p*<0.05; Fig. 4F) were significantly increased after the onset of hyperglycemia. On the other hand, EX-4 treatment reduced the levels of TLR4 (F(1,23) = 19.8464, *p*<0.001; Fig. 4A), COX1 (F(1,23) = 48.8869, *p*<0.001; Fig. 4B), COX2 (F(1,23) = 16.1276, *p*<0.001; Fig. 4C), NF-κB (F(1,23) = 46.8688, *p*<0.001; Fig. 4D), and CD45 (F(1,23) = 85.5516, *p<*0.001; Fig. 4E), and increased the MnSOD level (F(1,23) = 37.8035, *p*<0.001; Fig. 4G). In addition, EX-4 treatment reduced the COX2 (F(1,23) = 31.9798, *p*<0.001; Fig. 4C), NF-κB (F(1,23) = 8.5548, *p*<0.01; Fig. 4D), and CD45 (F(1,23) = 6.6012, *p*<0.05; Fig. 4E) levels against hyperglycemia, and COX1 (F(1,23) = 19.7972, *p*<0.001; Fig. 4B) and NF-κB (F(1,23) = 8.0, *p*<0.05; Fig. 4D) against intrahippocampal LPS injection.

After the MWM behavior task (day 39), the results of western blot analysis indicated that LPS treatment significantly increased the levels of COX2 (F(1,23) = 8.4511, *p*<0.05; Fig. 5C), NF-κB (F(1,23) = 5.3062, *p*<0.05; Fig. 5D), and CD45 (F(1,23) = 20.9074, *p*<0.01; Fig. 5E). Chronic hyperglycemia also significantly increased the levels of COX1 (F(1,23) = 45.9375, *p*<0.001; Fig. 5B), COX2 (F(1,23) = 25.6371, *p*<0.01; Fig. 5C), NF-κB (F(1,23) = 11.1632, *p*<0.01; Fig. 5D), and CD45 (F(1,23) = 7.8938, *p*<0.01; Fig. 5E). On the other hand, continuous EX-4 treatment significantly decreased the levels of COX1 (F(1,23) = 77.0667, *p*<0.001; Fig. 5B), COX2 (F(1,23) = 13.8062, *p*<0.01; Fig. 5C), NF-κB (F(1,23) = 4.7481, *p*<0.05; Fig. 5D), and CD45 (F(1,23) = 21.4143, *p<*0.001; Fig. 5E), and increased the MnSOD level (F(1,23) = 98.0837, *p*<0.001; Fig. 5G). Continuous EX-4 treatment reduced the levels of COX1 (F(1,23) = 44.2042, *p*<0.001; Fig. 5B), COX2 (F(1,23) = 14.3844, *p*<0.01; Fig. 5C), NF-κB (F(1,23) = 4.5689, *p*<0.05; Fig. 5D), and CD45 (F(1,23) = 9.8604, *p*<0.01; Fig. 5E) against hyperglycemia and the CD45 level (F(1,23) = 20.9074, *p*<0.001; Fig. 5E) against intrahippocampal LPS injection.

These results suggested that both chronic hyperglycemia and acute intrahippocampal injection induced the activation of NF-κB-related inflammatory responses. Furthermore, acute intrahippocampal LPS injection did not enhance the activation of the related NF-κB inflammatory responses in the hyperglycemic mice. However, EX-4 treatment not only inhibited the inflammatory-related proteins but also increased the MnSOD level. Therefore, EX-4 treatment induced the effects of both anti-oxidation and anti-inflammation.

### Intrahippocampal LPS Injection Greatly Increased Astrocytes Activation and Apoptosis, and Decreased Monoamine Neurotransmitter Neurons in the mice with Hyperglycemia, but EX-4 Treatment Protected Against the Impairment

**Figure 6:**
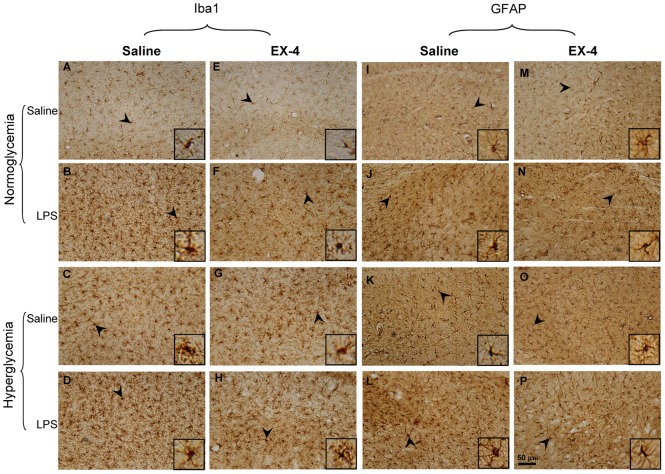
Immunohistochemical analysis of microglia and astrocytes in the mouse hippocampus. (A–H) Representative immunostainings of microglia by Iba1 antibody in the mouse hippocampus. (I–P) Representative immunostainings of astrocytes by GFAP antibody in the mouse hippocampus. Scale bar = 50 µm. Arrowheads indicated positive staining for activated microglia and astrocytes, which are magnified in the insets of each figure (*n = *3–5 per group).

The activation of glia plays an important role in the inflammation of progressive neurodegenerative diseases, including AD [24]. Therefore, the activation of microglia and astrocytes was examined in this study (Fig. 6 & Table 3). We found that hyperglycemia significantly increased the activation of microglia (F(1,252) = 12.6446, *p*<0.001; Fig. 6C & D) and astrocytes (F(1,252) = 40.092, *p*<0.001; Fig. 6K & L) as compared with normoglycemia. In addition, an acute intrahippocampal LPS injection also significantly increased the activation of microglia (F(1,252) = 732.32, *p*<0.001; Fig. 6B & D) and astrocytes (F(1,252) = 396.351, *p*<0.001; Fig. 6J & L) as compared with vehicle injection. EX-4 treatment reduced the activation of microglia (F(1,252) = 9.17, *p*<0.001; Fig. 6 F, G & H) and astrocytes (F(1,252) = 19.854, *p*<0.001; Fig. 6N, O & P) as compared with saline treatment. Furthermore, there was significant interaction of intrahippocampal LPS injection × EX-4 treatment for activated microglia (F(1,252) = 6.82, *p<*0.01; Fig. 6F), and hyperglycemia × intrahippocampal LPS injection (F(1,235) = 4.285, *p*<0.05; Fig. 6D) and intrahippocampal LPS injection × EX-4 (F(1,235) = 8.991, *p*<0.01; Fig. 6N) for activated astrocytes.

**Table 3:** The immunohistochemistry results in mice receiving different treatments.

	NSS	NSE	NLS	NLE	HSS	HSE	HLS	HLE
TH	105±2.9	123±2.1	79±0.3^b^ ***	125±3.6^b^ *** ^,^ c ***	35±2.4^a^ ***	58±2.6^a^ ***	28±1.5^a^ *** ^,^ b **	56±2.2^a^ *** ^,^ b *** ^,^ c ***
5-HT	53±2.1	55±1.7	14±0.7^b^ ** *	47±1.1^b^ * ^,^ c ***	9±0.6*^a^* ***	26±1.8^a^ *** ^,^ c **	7±0.4^a^ *** ^,^ b **	15±0.7^a^ *** ^,^ b *** ^,^ c ***
Iba1	24±3.3	27±2.2	85±3.6^b^ ***	73±2.7^b^ *** ^,^ c ***	35±2.9^a^ **	26±3.1	121±5.2^b^ ***	79±13.2^b^ *** ^,^ c ***
GFAP	13±1.3	11±1.7	52±4.5^b^ ***	31±3.2^b^ *** ^,^ c **	22±2.3^a^ **	20±1.6^a^ **	74±6.5^a^ *** ^,^ b **	48±2.5^c^ **
Caspase 3	12±2.2	13±2.1	135±4.3^b^ ***	114±5.3^b^ *** ^,^ c ***	51±4.6^a^ ***	55±2.1^a^ ***	229±1.8^a^ *** ^,^ b ***	187±1.4^c^ ***

Each value represents the mean ± SEM (n = 3–5 for each group).

a Normoglycemia compared with hyperglycemia.

b Saline compared with LPS.

c Saline compared with EX-4.

N, Normoglycemia; H, Hyperglycemia; L, LPS; S, Saline; E, EX-4.

* *p*<0.05;

** *p*<0.01;

*** *p*<0.001.

**Figure 7:**
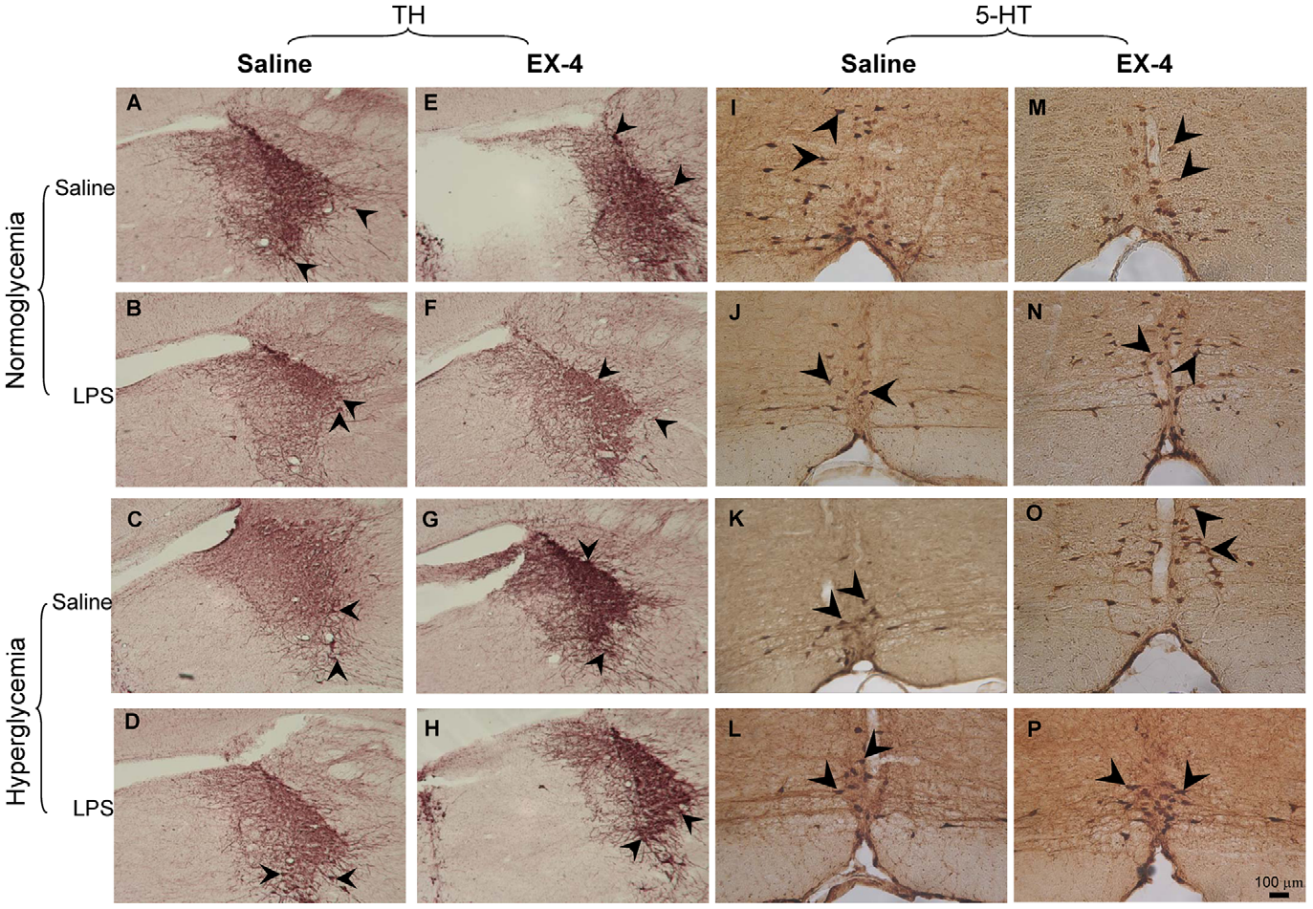
Immunohistochemical analysis of noradrenergic and serotonergic neurons. (A–H) Representative immunostainings of noradrenergic neurons in the locus coeruleus (LC) region. (I–P) Representative immunostainings of serotonergic neurons in the Raphe nucleus. Scale bar = 100 µm. Arrowheads indicate positive staining signals (*n = *3–5 per group).

Previous studies have shown that many neurochemical abnormalities of the cholinergic, noradrenergic, and serotonergic systems occur in the brains of AD patients [25], [26]. The effects of treatment on cholinergic (choline acetyltransferase immunoreactive, ChAT-ir), noradrenergic (tyrosine hydroxylase immunoreactive, TH-ir), and serotonergic (serotonin immunoreactive, 5-HT-ir) neurons in the mouse brain were then analyzed with immunohistochemistry (Fig. 7 & Table 3).

For the cholinergic neurons, no effect was identified in the mice with hyperglycemia, those that received intrahippocampal LPS injections, and those that received EX-4 treatment in the medial septum, vertical diagonal band of Broca, and horizontal diagonal band of Broca regions (data not shown). For noradrenergic TH-ir neurons in the locus coeruleus (LC), significant differences were observed due to intrahippocampal LPS injection (F (1,129) = 24.1999, *p*<0.001; Fig. 7B & D), hyperglycemia (F (1,129) = 1240.087, *p*<0.001; Fig. 7C & D), and EX-4 treatment (F(1,129) = 233.7521, *p*<0.001; Fig. 7F & H). Furthermore, there were significant interactions of hyperglycemia × intrahippocampal LPS injection (F(1,129) = 5.8661, *p*<0.05), intrahippocampal LPS injection × EX-4 (F(1,129) = 24.9438, *p*<0.001), and hyperglycemia × intrahippocampal LPS injection × EX-4 (F(1,129) = 13.0401, *p*<0.001). For 5-HT-ir neurons in the Raphe nucleus, significant differences were also observed due to intrahippocampal LPS injection (F(1,130) = 290.1053, *p*<0.001; Fig. 7J & L), hyperglycemia (F(1,130) = 1008.321, *p*<0.001; Fig. 7K & L), and EX-4 treatment (F(1,130) = 284.2575, *p*<0.001; Fig. 7N & P). There were also significant interactions of hyperglycemia × intrahippocampal LPS injection (F(1,130) = 90.0179, *p*<0.001), intrahippocampal LPS injection × EX-4 (F(1,130) = 41.0257, *p*<0.001), hyperglycemia × EX-4 (F(1,130) = 8.2344, *p*<0.01), and hyperglycemia × intrahippocampal LPS injection × EX-4 (F(1,130) = 123.4330, *p*<0.001). Therefore, these neurochemical results suggested that acute intrahippocampal LPS injection largely enhanced the deficits in the noradrenergic and serotonergic neurons in the hyperglycemic mice. However, EX-4 treatment protected against the deficits in the noradrenergic neurons of the LC region and the serotonergic neurons of the Raphe nucleus.

**Figure 8:**
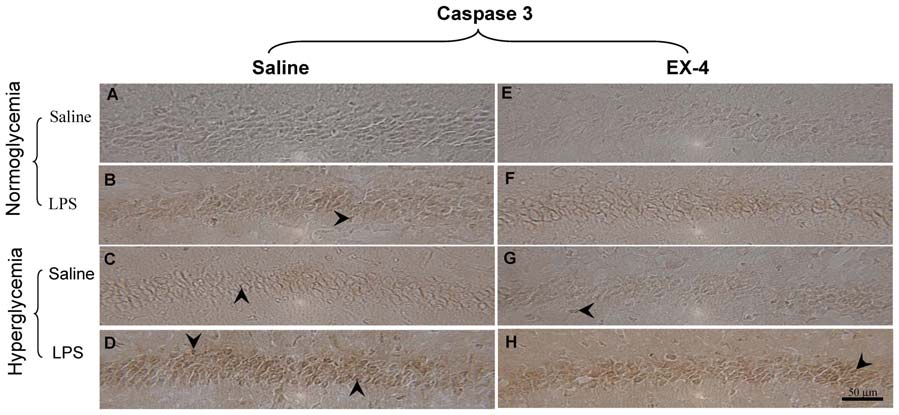
Immunohistochemical analysis of apoptosis in the mouse hippocampus. (A–H) Representative immunostainings of caspase 3 are shown in the CA1 subregion of the hippocampus. Scale bar = 50 µm. Arrowheads indicate positive staining signals (*n = *3–5 per group).

We further examined whether neuronal apoptosis was present in the CA1 subregion of the hippocampus of the hyperglycemia or/and an acute intrahippocampal injection groups (Fig. 8A-H). The quantitative results are also summarized in Table 3. We found that the levels of apoptosis were significantly increased in the mice that received an intrahippocampal LPS injection as compared with those that received vehicle injection (F (1,201) = 645.61, *p*<0.001; Fig. 8B). Furthermore, the apoptotic neurons were also found to be significantly increased in the hyperglycemic mice as compared with the normoglycemic mice (F(1,201) = 136.77, *p*<0.001; Fig. 8C & D). EX-4 treatment significantly prevented neuronal apoptosis (F(1,201) = 8.04, *p<*0.01; Fig. 8F–H). In addition, intrahippocampal LPS injection enhanced apoptosis in the hyperglycemic mice as compared with the normoglycemic mice (F(1,201) = 16.12; *p*<0.001; Fig. 8D). However, EX-4 treatment attenuated the impairment resulting from intrahippocampal LPS injection (F (1,201) = 10.98, *p*<0.01; Fig. 8H). Therefore, these results showed that acute intrahippocampal LPS injection greatly increased astrocytes activation and apoptosis and induced deficits in the noradrenergic and serotonergic neurons in the hyperglycemic mice. However, EX-4 treatment protected against these impairments.

## Discussion

In this study, we aimed to elucidate the mechanisms of chronic hyperglycemia and/or acute intrahippocampal LPS injection in the progression of the cognitive dysfunction. In addition, the neuroprotective effects of the EX-4 against hyperglycemia and/or acute intrahippocampal LPS damage were also evaluated in this study.

First, mice with STZ-induced diabetes developed hyperglycemia and hypoinsulinemia during the entire experimental period, which was consistent with recent reports [27], [28]. At the onset of hyperglycemia, the levels of IL-1β and inflammatory responses such as TLR4, COX1, COX2, NF-κB, CD45, and iNOS were increased in the hippocampus. In addition, the persistent hyperglycemia also induced cognitive dysfunction associated with increasing levels of COX1, COX2, NF-κB, CD45, microglia/astrocytes activation, and apoptosis in the hippocampus and decreasing levels of noradrenergic neurons in the LC and serotonergic neurons in the Raphe nuclei. An *in vitro* study showed that high glucose induced apoptosis through activation of caspase 3 [29]. Previous evidence has also suggested that hyperglycemia induces NF-κB activation as a result of increased levels of IL-1β [30], [31], [32]. Evidence also suggests that the interaction between IL-1β and NF-κB could turn the innate immunity into the driving force in cognitive dysfunction pathogenesis [33]. Recent study further showed that the positive feedback mechanism of NF-κB-mediated COX-2 amplified the pathologic levels [34]. Furthermore, the NF-κB pathway is a critical molecular system in the pathologic induction of brain inflammation, which leads to the metabolic syndrome [35]. Much evidence has suggested that long-standing type I diabetic patients are usually characterized as having cognitive impairment and dementia [36], [37]. Therefore, the elevated levels of NF-κB-related inflammatory responses may be responsible in a major way for the cognitive impairment in the hyperglycemic mice.

Our results also demonstrated that intrahippocampal LPS injection transiently increased the levels of IL-1β in the peripheral/central system and the levels of TLR4, COX1, COX2, CD45, and NF-κB in the hippocampus. In addition, acute intrahippocampal LPS injection resulted in cognitive dysfunction associated with long-term increases in the levels of COX2, CD45, NF-κB, microglia/astrocytes activation, and apoptosis in the hippocampus and decreases in the cognitive-related neurotransmission systems, noradrenergic and serotonergic neurons. Previous study has suggested that IL-1β plays an important role in mediating LPS-induced brain injury [38]. Recent evidence has also shown that the central nervous system inflammation triggered by infectious agents might lead to cognitive impairments [39], and several studies further suggest that the NF-κB pathway plays a crucial role in neuroinflammation-related neurodegenerative disorders such as AD [40], [41]. Therefore, our data revealed that the NF-κB related inflammatory responses might be a critical factor in the impairment of the spatial learning and memory resulting from intrahippocampal LPS injection or hyperglycemia.

Furthermore, we found that intrahippocampal LPS injection transiently enhanced the levels of IL-1β in the hippocampus and chronically exacerbated the impairment of spatial learning and memory associated with highly-induced astrocytes activation and apoptotic levels in the hippocampus and decreased noradrenergic neurons in the LC region and serotonergic neurons in the Raphe nuclei of the hyperglycemic mice. In this study, acute intrahippocmapal LPS injection exacerbated cognitive dysfunction in the hyperglycemic mice. Recent evidence also shows that inflammation induces acute exacerbations of dementia in aged patients [42]. Much evidence has shown that astrocytes play a role in the pathology of AD [43], [44], [45]. In addition, it has also been pointed out that the noradrenergic-related effects on astrocytes play an important role in the consolidation of long-term memory [46], [47]. Evidence further points out that astrocytes activation induces the production of caspase 3 in AD [48], [49]. Therefore, our data showed that chronic hyperglycemia associated with an acute intrahippocampal LPS injection exacerbated progression of the cognitive dysfunction, which could be attributed to the large increases in astrocytes activation-related responses in the hippocampus.

During continuous EX-4 treatment, we found that EX-4 decreased blood glucose and increased the insulin level in the hyperglycemic mice, which is consistent with some previous studies [50], [51]. In addition, in this study, EX-4 treatment also transiently increased IL- 1β in the normoglycemic mice and decreased IL-1β in the hyperglycemic mice. Previous study suggested that EX-4 increases the IL-1β level against apoptosis [52]. Evidence also shows that EX-4 inhibits iNOS expression via a decrease in IL-1β [53]. Therefore, the transient effects of EX-4 may be against apoptosis via increasing the IL-1β level in normoglycemia and iNOS via an inhibited IL-1β level in hyperglycemia. Furthermore, our study showed that EX-4 protected against the impairment of spatial reference learning and memory in mice with hyperglycemia and/or those receiving an intrahippocampal LPS injection. EX-4 treatment transiently reduced the levels of COX1 and NF-κB against intrahippocampal LPS injection, while continuous EX-4 treatment chronically reduced the levels of inflammatory related factors such as COX1, COX2, CD45, and NF-κB against hyperglycemia. EX-4 treatment also increased the anti-oxidant capacity during the experimental procedure. Furthermore, continuous EX-4 treatment protected against the reduction in noradrenergic neurons and serotonergic neurons, decreased astrocyte/microglia activation, and apoptosis in the hippocampus against intrahippocampal LPS injection. Recent studies further suggested that EX-4 treatment attenuates the inflammation and oxidative stress against acute ischemia [54], [55]. Evidence also suggests that short-term therapy with non-steroidal anti-inflammatory drugs (NSAID) fails to delay cognitive decline in patients with mild-to-moderate AD [56]. However, long-term NSAID therapy usually induces gastrointestinal toxicity [57]. Therefore, these results suggest that EX-4 treatment is an effective and safe treatment to protect against cognitive dysfunction via anti-oxidation and anti-inflammation.

In conclusion, our results suggest that the activation of the NF-κB-related inflammatory responses induced cognitive dysfunction in both the hyperglycemia and intrahippocampal LPS injection groups. However, acute intrahippocampal LPS injection exacerbated the progression of cognitive dysfunction through large increases in astrocytes activation-related responses in the hyperglycemic mice. Furthermore, EX-4 treatment protected against the impairment of cognition resulting from hyperglycemia and/or intrahippocampal LPS injection. Therefore, these findings revealed that EX-4 might be considered as a potential adjuvant neuroprotective agent for neurodegenerative diseases.

## Materials and Methods

### Animals

A total of 200 male C57BL/6J mice (6–8 weeks old) were purchased from the National Center for Laboratory Animals Sciences (Taipei, Taiwan). The mice were randomly divided into eight groups, with 25 animals in each group: (i) normoglycemia/saline vehicle in CA1/saline treatment; (ii) normoglycemia/saline vehicle in CA1/EX-4 treatment; (iii) normoglycemia/LPS in CA1/saline treatment; (iv) normoglycemia/LPS in CA1/EX-4 treatment; (v) hyperglycemia/saline vehicle in CA1/saline treatment; (vi) hyperglycemia/saline vehicle in CA1/EX-4 treatment; (vii) hyperglycemia/LPS in CA1/saline treatment; and (viii) hyperglycemia/LPS in CA1/EX-4 treatment. The mice were housed at 20–25°C and 60% relative humidity under a 12-h light/dark cycle, and food and water were made available ad libitum. All experiments were performed during the light phase between 7 AM and 7 PM. All experimental procedures involving animals were performed according to the guidelines established by the Institutional Animal Care and Use Committee of National Taiwan Normal University, Taipei, Taiwan.

### Experiment Timeline

After one week of adaptation to the home cage, mouse body weight and blood glucose were measured on days 1, 10, 32 and 39 (Fig. 1). Streptozotocin (STZ; Sigma, Saint Louis, MO, USA) or control was injected into the mice on day 1 after measurement of body weight and blood glucose. On day11, either EX-4 (10 µg/Kg; Sigma) or vehicle (normal saline) was administered by a once-daily intraperitoneal injection for 28 days in mice. An intrahippocampal CA1 injection of LPS or saline vehicle was conducted 30 min after EX-4 application. Mice were sacrificed for western blot and enzyme-linked immunosorbent assay (ELISA) analyses on day 12. Subsequently, pretraining, training, testing and probe in the Morris water maze were performed on days 32–38. Mice were sacrificed for immunohistochemistry, western blot and ELISA analyses on day 39.

### Generation of Hyperglycemic Mice

Mouse blood samples were obtained by tail prick and glucose levels were measured using a commercial glucometer (Accu-Check Active; Roche, Mannheim, Germany) before hyperglycemia induction. STZ (200 mg/kg in 0.1 ml sodium citrate buffer, pH4.5; Sigma) was intraperitoneally injected into non-fasting mice within 15 min to induce hyperglycemia as previous described [58]. The normoglycemic groups were injected with an equivalent volume of citrate buffer. Ten days after STZ treatment, subjects who failed to develop hyperglycemia (defined as a blood glucose concentration >200 mg/dl) were excluded from this study.

### LPS Treatment

After EX-4 or saline intraperitoneal injection, normoglycemic and hyperglycemic subjects were infused with 1 µl of LPS (4 µg/µl; Calbiochem, CA, USA) or vehicle (normal saline) into the CA1 subregion of the hippocampus. First, each mouse was anesthetized with avertin (0.4 g/kg body weight; Sigma) and then placed in a stereotaxic instrument (DKI-900, David Kopf Instruments, CA, USA). An incision was made in the scalp and a hole was drilled into the skull over the injection site. A 30-gauge-needle was then placed into the dorsal hippocampus. The coordinates for the anterior–posterior (from bregma), medial–lateral (from midline), and dorsal–ventral (from surface of the skull) axes were −2.3, ±2.5, and −1.5 mm, respectively. Bilateral intrahippocampal infusion was administered via a 10.0-µl Hamilton microsyringe with a 30-gauge needle fitted to the arm of the stereotaxic instrument. About 1.0 µl of LPS or vehicle was slowly infused at a rate of 0.35 µl per minute. After an additional 5 min to ensure adequate diffusion, the needle was slowly retracted from the animal. After recovery from surgery, mouse body weight and blood glucose were measured, and a series of cognitive tasks were then performed.

### Morris Water Maze (MWM) Task

Mouse spatial learning and memory were evaluated using a conventional MWM, a device commonly used for studying cognitive deficits in APP transgenic mice [59]. The water maze apparatus consisted of a circular pool (1.0 m diameter and 0.47 m high) made of white plastic; the pool was filled to a depth of 40 cm with water (24–25°C) and made opaque by the addition of nontoxic white paint. During conventional MWM training, an escape platform (10 cm in diameter) made of white plastic, with a grooved surface for better grip, was submerged 1.0 cm underneath the water level. Cues of various types provided distal landmarks in the testing area of the room. The swimming path of the mouse during each trial was recorded by a video camera suspended 2.5 m above the center of the pool and connected to a video tracking system (EthoVision, VA, USA). On the day prior to spatial training, all mice underwent pre-training in order to assess their swimming ability and to acclimatize them to the pool. Each mouse was first placed on a visible platform located at the center of the pool and allowed to stay there for 20 sec. In the following three 60-sec trials, the mouse was released into the water facing the wall of the pool from semi-randomly-chosen cardinal compass points. If the mouse failed to swim to the platform or stay on it for 20 sec, it would be placed on the platform by an experimenter. The mice were given a 4-day training session consisting of four 60-sec training trials (inter-trial interval: 20–30 min) per day. The hidden platform was always placed at the same location of the pool (Northeast quadrant) throughout the training period. During each trial, from quasi-randomly chosen cardinal compass points, the mouse was released into the water facing the pool wall. After climbing onto the platform, the mouse was allowed to rest on it for 20 sec. After the last training trial, all mice were given three testing trials to assess the time taken to climb onto the hidden platform. Twenty-four hours after the last testing trial, all mice were given three probe trials to evaluate the retrieval of the spatial memory regarding the platform.

### Immunohistochemistry

Immediately after the water maze test, mice (n = 6 per group) were anesthetized (avertin; 0.4 g/kg body weight) and transcardially perfused with 4% paraformaldehyde in phosphate-buffered saline (PBS). Mouse brains were removed and post-fixed with 4% paraformaldehyde for 4 hr and cryo-protected with 10% sucrose for 1 hr followed by 20% sucrose for 2 hr, then placed in 30% sucrose in PBS for 2 days, followed by continuous serial cryostat sectioning at 30 µm on a crystat-microtome (CMS3050S, Leica Microsytems, Nussloch, Germany). Histology and immunohistochemistry were performed to assess the location of the infusion needle tip and to identify any anatomical and neurochemical abnormalities induced by the STZ, LPS, and EX-4 treatments. Specific antibodies were used to assess the neurotransmitters by rabbit anti-ChAT polyclonal antibody (choline acetyltransferase, cholinergic specific, 1∶500; Millipore, MA, USA), rabbit anti-TH polyclonal antibody (tyrosine hydroxylase, noradrenergic-specific, 1∶1000; Millipore), and rat anti-serotonin monoclonal antibody (5-HT, serotonergic-specific, 1∶100; Millipore). Neuroinflammatory response was identified by rabbit anti-Iba1 polyclonal antibody (1∶1000; Wako, Osaka, Japan) and rabbit anti-GFAP polyclonal antibody (1∶1000; Millipore); cell apoptosis was analyzed by rabbit anti-caspase3 antibody (1∶30; Millipore).

Free-floating sections were used for immunohistochemistry and immunofluorescence. In brief, blocking of the endogenous peroxidase of sections was performed by incubation with 3% H_2_O_2_. After washing with PBS three times (10 min/wash), nonspecific epitopes of sections were then blocked by incubation in 5% normal goat serum or normal rat serum and 0.1% Triton X-100 in PBS for 1 hr. Sections were incubated with primary antibodies overnight at room temperature, washed with PBS, and incubated in the secondary antibodies (1∶200 dilution in blocking solution, Vector Laboratories, CA, USA) for 1 hr, and finally incubated in an avidin-biotin complex for 1 hr at room temperature. The reaction was developed using a diaminobenzidine (DAB) kit (Vector). All sections were mounted on gelatin-coated slides and cover-slipped for light microscopy. The images obtained were loaded in a research-based digital image analysis software (Image Pro Plus, Media Cybernetics, MD, USA) and the threshold intensity was set manually to keep constant for all photomicrographs as the number of DAB pixels was counted. Pixel counts were taken as the average from three adjacent sections per animal, and the data were presented in Table 3.

### Western Blot Analysis

Proteins were extracted from the whole hippocampus of the mice (n = 6 per group). The protein concentration was determined using a bicinchoninic acid (BCA) protein assay kit (Pierce, USA). Proteins (50 µg) were separated by SDS-PAGE and transferred to membranes. The primary antibodies used were goat anti-TLR4 polyclonal antibody (1∶200; SANTA CRUZ, CA, USA), rabbit anti-MnSOD polyclonal antibody (1∶1000; Millipore), rabbit anti-NF-κB polyclonal antibody (1∶2000; Chemicon), rabbit anti-CD45 polyclonal antibody (1∶500; SANTA CRUZ), rabbit anti-COX-2 polyclonal antibody (1∶1000; Millipore), rabbit anti-iNOS polyclonal antibody (1∶500; Cayman, MI, USA), mouse anti-COX-1 monoclonal antibody (1∶500; Chemicon) and mouse monoclonal β-actin antibody (1∶2000; Chemicon); and the secondary antibodies used were anti-rabbit IgG HRP-linked antibody (1∶10000; Cell Signaling, USA), anti-goat IgG HRP-linked antibody(1∶5000; Sigma), and anti-mouse IgG HRP-linked antibody (1∶10000; Cell signaling). The specific antibody–antigen complex was detected using an enhanced chemiluminescence detection system (Amersham Pharmacia Biotech, MA, USA). The intensity of the signals of the western blots was quantified using an LAS-3000 chemiluminescence detection system (Fujifilm, Tokyo, Japan) and was expressed as the ratio relative to β-actin protein.

### Enzyme-linked Immunosorbent Assay (ELISA)

Blood samples were collected from the retro-orbital plexus of anesthetized mice and centrifuged at 2,000×g for 15 min at 4°C on days 12 and 39 (n = 6 for each group). Plasma was collected and stored at −80°C until use. Frozen hippocampus was homogenized and centrifuged as described previously [60]. The levels of IL-1β in the hippocampus and plasma were measured using IL-1β ELISA kits (R&D Systems, MN, USA), and the insulin level in the plasma was determined using an Ultra Sensitive Mouse Insulin ELISA kit (Crystal Chem, IL, USA) following the manufacturer’s instructions.

### Data Analysis

Data were analyzed by three-factor analysis of variance (ANOVA) by group (normoglycemia and hyperglycemia), intrahippocampal infusion (LPS and saline), and intraperitoneal treatment (EX-4 and saline), followed by Student’s *post hoc t*-testing in order to compare the effects of all treatments. The results were expressed as the mean ± SEM. Differences were considered statistically significant if *p*<0.05.
